# Signatures of Crested Ibis MHC Revealed by Recombination Screening and Short-Reads Assembly Strategy

**DOI:** 10.1371/journal.pone.0168744

**Published:** 2016-12-20

**Authors:** Liao Chang, Shiyang He, Danqing Mao, Yuanhong Liu, Zijun Xiong, Dongke Fu, Bo Li, Shuguang Wei, Xun Xu, Shengbin Li, Hui Yuan

**Affiliations:** 1 College of Medicine and Forensics, Xi’an Jiaotong University, Xi’an, Shaanxi, China; 2 BGI-Shenzhen, Shenzhen, Guangdong, China; 3 College of Animal Science and Technology, Northeast Agricultural University, Harbin, Heilongjiang, China; Virginia Tech Virginia, UNITED STATES

## Abstract

Whole-genome shotgun (WGS) sequencing has become a routine method in genome research over the past decade. However, the assembly of highly polymorphic regions in WGS projects remains a challenge, especially for large genomes. Employing BAC library constructing, PCR screening and Sanger sequencing, traditional strategy is laborious and expensive, which hampers research on polymorphic genomic regions. As one of the most highly polymorphic regions, the major histocompatibility complex (MHC) plays a central role in the adaptive immunity of all jawed vertebrates. In this study, we introduced an efficient procedure based on recombination screening and short-reads assembly. With this procedure, we constructed a high quality 488-kb region of crested ibis MHC that consists of 3 superscaffolds and contains 50 genes. Our sequence showed comparable quality (97.29% identity) to traditional Sanger assembly, while the workload was reduced almost 7 times. Comparative study revealed distinctive features of crested ibis by exhibiting the COL11A2-BLA-BLB-BRD2 cluster and presenting both ADPRH and odorant receptor (OR) gene in the MHC region. Furthermore, the conservation of the BF-TAP1-TAP2 structure in crested ibis and other vertebrate lineages is interesting in light of the hypothesis that coevolution of functionally related genes in the primordial MHC is responsible for the appearance of the antigen presentation pathways at the birth of the adaptive immune system.

## Introduction

The major histocompatibility complex (MHC) is involved in immunity defenses and mate choice in all jawed vertebrates [1, 2]. Increasing evidence has indicated that individuals make mating choices with respect to MHC. In order to produce offspring with stronger immune system, females tend to mate with males who carrying genetically different alleles or some specific advantage alleles [3–5]. Characterized by high polymorphism, large allelic differences, repeat-rich and GC-rich, MHC is recognized as one of the most complex genomic regions. Although whole-genome shotgun (WGS) sequencing has become a routine approach to collect data for *de novo* genome assembly, it remains a challenge to construct MHC regions with the WGS method.

The traditional solution for MHC construction has mainly depended on 1) construction of a bacterial artificial chromosome (BAC) library; 2) target clone screening by PCR or hybridization; and 3) sub-clone sequencing with long-read sequencing technology, such as Sanger, Roche 454 or PacBio [6–9]. Choosing enough single clones to fill plates in BAC library construction and PCR-based screening of target clones make traditional solutions laborious. Moreover, hybridization-based screening shows high false positive rates [10]. These shortages make traditional strategies as well as genome finishing efforts less productive, leaving the MHC region unsolved in most sequenced species.

The available data show that MHC organization varies among vertebrate lineages. Mammal MHCs generally span several megabases as a single complex [1, 11, 12] and fishes have highly dispersed MHC genes [13]. Studies on avian MHCs have mainly focused on Galliformes. The representative chicken MHC contains 2 genetically unlinked clusters in GGA16: MHC-B and Rfp-Y [14]. The reported MHC-B region is highly compact [15]. Although other Galliformes birds share similar MHC characteristic with chicken [16, 17], the recently reported zebra finch (Passerines) MHC genes are distributed on at least 3 chromosomes [18, 19]. These data indicate that the genomic organization of avian MHCs may vary among different lineages.

Crested ibis in Pelecaniformes is a recently near-extinct and rebirth bird species, which provides a successful model of human efforts on species conservation. As a result, all current individuals originated from 2 breeding couples discovered in 1981 at Yangxian, China [20]. However, a recent study reported that the present crested ibis population exhibits low genetic polymorphism at the genomic level as well as the antigen binding region of the MHC class II β gene, which may result from high inbreeding and cause potential defective immunity in the bird population [21]. A complete MHC assembly of crested ibis is urgently needed for better conservation of this species and is also helpful in understanding the structural variation and evolution process of MHC.

A recently reported recombination method relies on recombineering to fish out target fosmid clones from pools and thereby circumvents the laborious steps of plating and screening thousands of individual clones with traditional solutions [22]. In this study, based on the genomic fosmid library pools, we adopted recombination screening followed by clone-based sequence and assembly (CSA) to assemble the crested ibis MHC.

## Results

### MHC-containing clone screening

**Fig 1:**
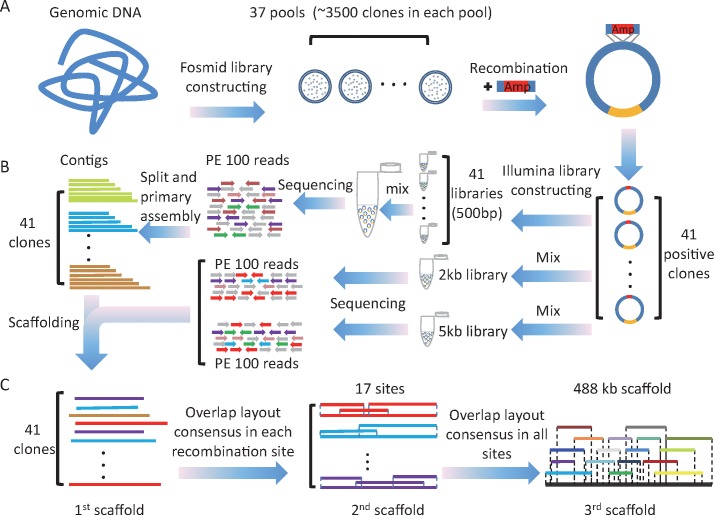
Overview of recombination-based CSA strategy. (A) Fosmid library construction and target clones isolation. ~40 kb fragments of genomic DNA were ligated to the pCC2Fos vector. Then these loaded vectors were transduced into *E*. *coli* clones, which were divided into 37 pools. After screening the pools by PCR, recombinant cassettes were used to isolate target clones from positive pools. (B) Illumina library construction and sequencing. One small insert size library was constructed for each clone and a large insert size library was constructed with a mixture DNA of all clones. (C) Step by step assembly strategy of the target region. Each clone was sequenced and assembled separately to get the first class scaffold. First class scaffolds derived from the same recombinant site were assembled to second class scaffold by overlap relationship. Second class scaffolds from all sites were finally assembled to obtain the sequence of the target region.

To obtain the MHC region, a ~10-fold genome equivalent fosmid library containing 37 pools was constructed. Subsequently, referring to the incomplete genomic resources [23], 17 target sites were identified to isolate fosmid clones that contain MHC sequences (see the Methods section for detailed procedures). At each target site, a pair of specific screening primers (S1 Table) was designed to screen the pools and 4~10 positive pools were obtained. Also, based on the sequence of PCR production in the positive pool, a recombinant cassette was designed for each target site (S2 Table). Lastly, after electroporation, recombination reaction and ampicillin plate screening, 78 target clones were fished out. All 78 clones were end-sequenced with vector primers and identical ones were excluded. Finally, 41 clones were left to construct the MHC region (Fig 1A).

### CSA

**Fig 2:**
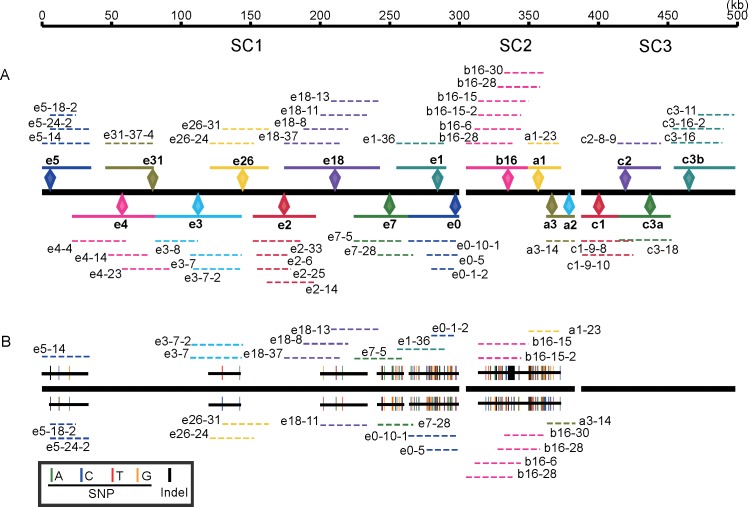
Assembly of the MHC region. (A) Reference assembly. Each color represents one target site. In each target site, the diamond indicates the position of the recombination cassettes; the dashed color line indicates the scaffold of each clone; the solid color line indicates the second class scaffold of each target site. The black bold line in the middle indicates the 3 super-scaffolds of the MHC. Recombinant sites C3a and C3b came from the same recombination cassette. (B) Partial haplotype separation. Paired black lines in addition to the super-scaffolds indicate the separated regions; short bars on paired black lines indicate the SNP or Indel differences between 2 chromosomes; the dashed colored lines indicate the clones used for determining the haplotypes.

After expansion, fosmid DNA of each selected clone was isolated. One 500 bp insert size library was constructed for each clone and 2 large insert size (2 kb and 5 kb) libraries were constructed with the pool of 48 fosmid clones. Sequencing was performed on an Illumina Hiseq 2000 platform with the 100 bp paired-end sequencing strategy (Fig 1B). After filtering the vector contamination, *ampR* gene sequences on selection cassettes and low quality reads, 480 Mb of clean data were obtained and the average sequence coverage per clone was ~250-fold. First, each clone was assembled to a first class scaffold individually and the size of the assembled scaffolds ranged from 28 kb to 47 kb with a peak at 39 kb (S1 Fig and S3 Table). Second, clones derived from the same recombinant cassette were assembled to a second class scaffold according to overlap relationships (Fig 1C). Finally, the second class scaffolds of 17 target sites were used to construct super-scaffolds SC1, SC2 and SC3 based on overlap relationships, and 3 super-scaffolds totally spanned 488.21 kb (Fig 2A).

By comparing the overlapped clones, we further identified haplotypes in the ~174 kb clone region. At the SC1 region, 4 regions that spanned 114 kb in total were separated by 76 SNPs and 10 Indels. At the SC2 region, 137 SNPs and 14 Indels were identified. Three clones (b16-15-2, b16-15 and a1-23) represented the first haplotype and other 4 clones (b16-28, b16-6, b16-28-2 and a3-14) represented another haplotype (Fig 2B).

### Assessment of short-read assembly

**Fig 3:**
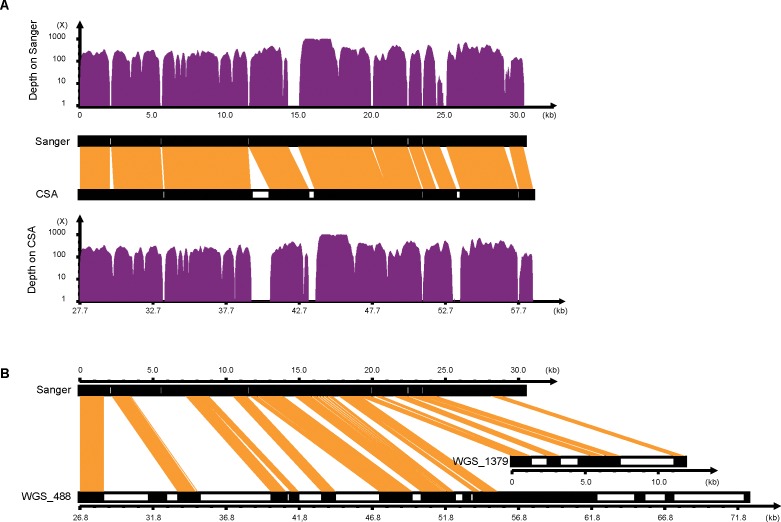
Assessment of CSA assembly. (A) Alignment between CSA assembly and Sanger assembly. (B) Alignment between comparisons of the WGS assemblies with the Sanger assembly. The orange blocks indicate the consistent sequences between the 2 strategies. Single-base depth was calculated by mapping the short-read to the Sanger and CSA assemblies, respectively.

To assess the local accuracy of CSA, we randomly sequenced a fosmid clone containing the BLB locus in the BF/BL region with the Sanger method (~20-fold coverage) and assembled it with phred-phrap-consed. Sanger and CSA assemblies were aligned using BLAT with a default parameter and the sequence identity reached 97.29% (Fig 3A), which was remarkably higher than that of WGS (50.96%; Fig 3B). Ten sequence differences between the Sanger and CSA assemblies were totally identified. Four of them were located in runs of Gs, and the rest were located in gap regions of the CSA or Sanger assembly. In summary, 6 and 4 potential misassemblies were validated in Sanger and CSA, respectively (Table 1). All information about these differences is detailed in S2 Fig.

**Table 1:** Assembly differences between Sanger and CSA.

No.	CSA start	CSA end	Sanger start	Sanger end	CSA description	Sanger description	Potential misassembly
**1**	29706	29892	2045	2145	Normal	Gap	Sanger
**2**	34393	34394	6540	6542	Low coverage	Normal	CSA
**3**	37374	37379	9522	9522	5bp insertion	Normal	CSA
**4**	38267	38274	10406	10406	7bp insertion	Normal	CSA
**5**	43327	43741	14237	14953	Gap	No coverage	Sanger
**6**	48699	48700	19910	20042	Low coverage	Gap	CSA
**7**	51049	51147	22390	22489	Normal	Gap	Sanger
**8**	53241	53241	24442	24443	Normal	1bp insertion	Sanger
**9**	53472	53684	24612	25074	Gap	Poor coverage	Sanger
**10**	57569	57769	28999	29366	Gap	Low coverage	Sanger

To eliminate incorrect assembly caused by potential chimeric clones and assess the overall accuracy, we used the mate pair reads of large insert-size libraries (2 kb, 5 kb, 10 kb and 20 kb) in the crested ibis WGS project [23] and mapped them to our MHC sequences. The pair-end relationship is presented in S3 Fig, which is well mapped and supports the validity of our assemblies. We also tried to determine the organization of 3 super-scaffolds, by considering all possible connections and looking for supporting evidences from WGS pair end reads. Interestingly, 16 mate pair reads (1 pair with 10 kb and 15 pairs with 20 kb insert size) showed that SC2 and SC3 were tightly linked within BF/BL region (S3 Fig). Long PCR was also used but failed to obtain informative products to fill the gap between SC2 and SC3.

### Annotation of crested ibis MHC

**Fig 4:**
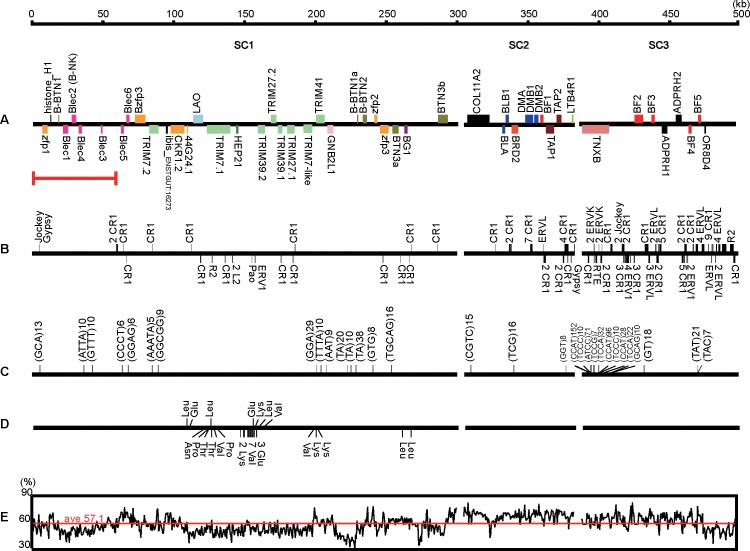
Gene organization and sequence features of the crested ibis MHC. (A) MHC gene map. Genes belong to the same family are indicated with the same color. Upper boxes indicate genes on a positive strand, and lower boxes indicate the opposite. The red bar indicates the MHC region that is first described in the present study. (B) Position of repetitive elements, LINEs and LTRs are represented by black bars. (C) Location of STRs. STRs with 2–5 bp repeat units are shown. (D) Location of tRNA loci. (E) Plot of local GC content in continuous 500 bp windows. The red line indicates the average GC content.

Genes, transposable elements (TEs), short tandem repeats (STRs) and tRNAs of the MHC region were annotated. Combining the homologous and *de novo* gene prediction approach, we identified 50 genes in the MHC region, corresponding to one gene per every 9.96 kb (Fig 4A). SC1 contains Blec genes, TRIM family, BTNs and BGs, SC2 ranges from COL11A2, class II genes to TAPs and LTB4R1, and SC3 represents TNXB, several class I genes and ADPRHs. Based on the nomenclature for chicken MHC, SC1 was classified into the extended BF/BL region [24], SC2 and SC3 were classified into the BF/BL region [25]. By mapping the RNA-Seq data [21] to MHC sequences, 29 genes were verified to be expressed in blood (S4 Table). In addition, 84 long interspersed nuclear elements (LINEs, 76 CR1, 3 Jocker, 2 L2, 2 R2 and 1 RTE), 32 long terminal repeats (LTRs, 19 ERVL, 6 ERV1, 4 ERVK, 2 Gypsy and 1 Pao; Fig 4B and S5 Table), 31 STRs (Fig 4C) and 30 tRNA loci (Fig 4D and S6 Table) were identified. In addition, using 500 bp non-overlapping sliding windows, the crested ibis MHC showed a high average GC content of 57.1% (Fig 4E).

### Comparative analyses of the avian MHC

**Fig 5:**
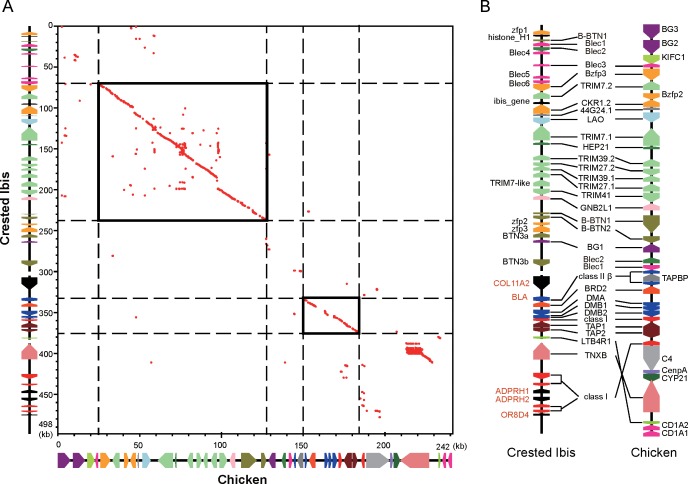
Comparative analyses of crested ibis and reported chicken MHC-B. (A) Dot matrix analysis of the crested ibis vs. the reported chicken MHC-B sequences. The red dot indicates that the sequences are homologous between 2 species at this position. Rectangles with a solid line indicate regions that demonstrate a high degree of synteny between the 2 birds. (B) Gene organization comparison. Genes in red indicate absence in reported chicken MHC-B sequence.

We compared the crested ibis MHC with reported chicken MHC-B and found that both gene family members and overall organization were similar between the 2 species. Particularly, aligning with PipMaker [26], 2 compact blocks, the TRIM cluster and BLB-BRD-DMA-DMB-BF-TAP1-TAP2 cluster, also showed high sequence identity between the 2 birds (Fig 5A). However, several genes, such as COLL11A2 and ADPRHs, were found in the crested ibis but absent in the reported chicken MHC-B sequence. Additionally, more copies of Blec and BF were found in the crested ibis than in the reported chicken MHC-B (Fig 5B). We also compared the TEs of MHCs between chicken and crested ibis. With the same annotation pipeline, 33 TEs (22 LINEs and 11 LTRs) were identified in the reported 242 kb chicken MHC-B region [15]. The crested ibis MHC showed a frequency of 0.23 TEs per kb, which was higher than that of the reported chicken MHC-B (0.13 TEs per kb) (S5 Table).

## Discussion

Featured by recombination isolation and a short-read assembly, the CSA strategy introduced in the study is much more efficient than traditional solutions. In the ~10-fold genomic fosmid library, we used ~230 PCR reactions to screen the positive pools and ~70 recombination reactions to fish out a ~488 kb region. While referring to previous reports [27, 28], ~2,000 PCR reactions or near 7-fold workload of CSA were needed if using 4D-PCR or other routine methods to screening the BAC library to obtain the same region. It is worth noting that the workload reduction rate of CSA increases along with an increase in the target region size (over several Mbs). This is because CSA scans and picks positive pools with low-depth sequencing instead of the PCR screening step used in traditional approaches. In addition, for long-term preservation, pooling thousands of clones together in a fosmid library is more convenient and safer than keeping individual clones in one well in a BAC library.

The comparison results showed that CSA and Sanger assembly shared a comparable number of potential local misassemblies at the inconsistencies, and the most notable errors between the 2 assemblies were short insertions or deletions caused by homopolymer size differences (S2 Fig and Table 1). These data suggested that CSA could achieve comparable accuracy with a traditional strategy and significantly improved the quality of highly polymorphic regions in WGS projects. The high quality CSA assembly may have benefited from the three factors. First, target region isolation and proper sequencing coverage can avoid a genomic sequencing bias. Second, CSA assembles the clone individually and therefore can avoid interference of large allelic differences. Lastly, complex genomic regions usually harbor internal repeats in a genome-wide context, while single clones reduce this complexity. In other words, the “complex region” is complex in a genome-wide context but could be relatively simple in an isolated single clone. Additionally, with a fosmid pooling sequencing strategy similar to CSA, the genome assembly quality of Oyster and Norway spruce has been significantly improved [29, 30].

**Fig 6:**
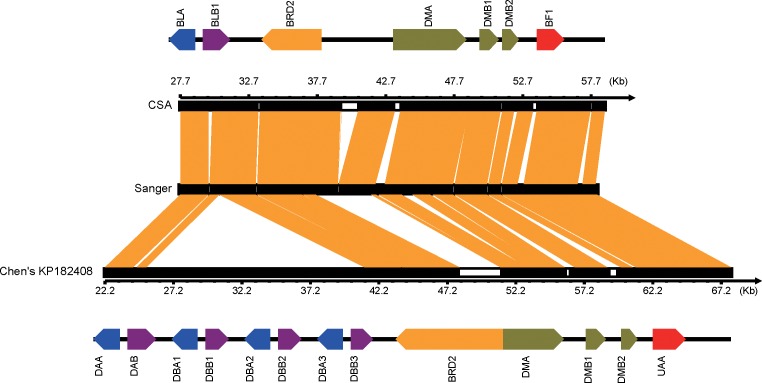
Gene organization and sequence feature comparison between CSA and Chen’s report in the Sanger assembly region. The black bars indicate the scaffold in 3 sequencing strategies and the white boxes stand for gap regions. The orange blocks between the 2 scaffolds indicate the concordant sequence. The top and bottom colorized bars indicate the genes on CSA and Chen’s scaffolds, respectively.

Recently, Chen and co-workers reported a partial MHC of crested ibis using a BAC library and 454 sequence approach (GenBank accession number: KP182407–KP182409) [31]. We compared the CSA with Chen's results from sequences and gene maps in common regions. Overall, the comparison showed that the sequence identity of the 2 methods in the extended BF/BL region and BF/BL region were 94.7% (SC1), 88.9% (SC2) and 82.1% (SC3), respectively. The well covered single base depth indicated the high quality of our CSA in the extended BF/BL region (S4A Fig). Relatively low identity in the BF/BL region between the 2 methods could be partly attributed to the difference between two haplotypes (S4B and S4C Fig). Furthermore, both CSA and Chen's results showed high consistency with the Sanger assembly in non-gap regions. One exception was the difference in copy numbers of the BLA/BLB pair. Only one pair was identified in CSA, while 4 tandem BLA/BLB (DBA/DBB) pairs were found in Chen's assembly (Fig 6). By Southern blotting, Taniguchi [32] totally found 3 haplotypes in the MHC class II region in the 5 founders of Japanese crested ibis, and the tandem number of BLA/BLB varied among haplotypes. Therefore, we inferred that our assembly and Chen's should be 2 different haplotypes. Lastly, the gene set and organization of CSA were highly consistent with Chen’s report, but further comparison showed some interesting differences (S4 Table). In the BF/BL region, 2 ADPRH gene loci were identified with CSA while 3 were identified in Chen’s, which might be caused by the gap in our assembly (S4C Fig). In the extended BF/BL region, a 2-kb BG gene was identified with CSA, while Chen's BG loci spanned over 30 kb with a large insertion in the same location but without significant homology (S4A Fig and S4 Table). Though potential minor misassembly may be included, differences between the 2 reports revealed a high degree of divergence between alleles. Furthermore, we noticed that Chen also failed to link two scaffolds in BF/BL region [31], thus we inferred that there may be a complex structure in this gap which increased the difficulty of amplification and sequencing.

It is a significant result that a new ~60 kb region containing 7 genes (1 zfp, 1 histone, 1 B-BTN1 and 4 Blec genes) was first reported at the Blec end of the crested ibis extended BF/BL region in our assembly (Fig 4A). The original chicken BF/BL sequence identified two different functional lectin-like genes, B-NK and B-lec. B-NK was found to be expressed in NK cell clones, thus closely related to NK receptor named NKR-P1 in mammals [25]. And the B-lec was most closely to human LLT1, the ligands of NKR-P1 [33]. In the annotated crested ibis Blec genes, the Blec2 gene belongs to the B-NK lineage, and others belong to B-lec lineage.

Beyond previous expectations, our results suggested that the size of the crested ibis MHC was larger than 500 kb. In addition, intensive SNPs and Indels between the 2 haplotypes in the BF/BL region (SC2) further suggested a high polymorphism between the alleles. Considering the history of a severe bottleneck, these combined data indicated that the reported relatively few MHC genetic polymorphisms in the crested ibis population might mainly present as a limited number of alleles, which has been observed in other bottlenecked species such as Seychelles warbler [34] and Arabian oryx [35].

For size, the crested ibis MHC is over twice of the reported compact chicken MHC-B in Galliformes. Comparative analyses also revealed some interesting signatures in the crested ibis MHC gene organization. First, the COL11A2-BLA-BLB-BRD cluster is found in the crested ibis MHC and gene duplications occurred as a unit with BLA/BLB pairs [32]. BLA gene is found in various avian species, but its genomic location varies from species to species. In the duck, a single BLA gene is located next to five BLB genes [36]. In the parrot, a BLA gene is adjacent to a BLB gene [37]. In the chicken, although located ~5 cM away from the BF/BL region [38], the single BLA has been mapped to the B locus by radiation hybrids and is considered as part of MHC-B [39]. Second, the odorant receptor (OR) gene is found adjacent to BF genes in the crested ibis MHC. In chicken, though located on the same chromosome, the OR gene is separated from MHC region by nucleolar organizer region [40]. Moreover, one B-NK and 2 Blec genes are located in the BF/BL region in chicken. In the crested ibis, B-NK and B-lec genes are located in the MHC region but away from BF/BL region. While in the zebra finch, B-NK and B-lec are located on the Z chromosome [19]. Finally, C4 and tapasin (TAPBP) gene are present in the reported MHC of chicken and many other vertebrates, but are not identified in our sequences. To verify whether these genes are actually present in the genome, we further used BLAST to search for homologues of chicken C4 and TAPBP gene in the crested ibis genome assembly (Accession: GCF_000708225.1). The best hit is a TAPBPL gene (GeneID:104009714) coding tapasin-related protein, which sharing only 34% identity with chicken TAPBP (ProteinID:BAF62997) in the 50% query cover region. For C4, the best hit is LOC104010873 (GeneID:104010873) coding complement C4-like protein, which sharing 42% identify with chicken C4 (ProteinID:BAF63007) in the 28% query cover region. Though failed to identify the C4 and TAPBP gene in crested ibis genome, we cannot rule out the possibility of low sequence conservation among the two species or C4/TAPBP located in the unfilled genome sequences gap. It is noteworthy that TAPBP is also not found in ducks [41]. These distinctive differences provide evidence for the fast evolution of avian MHCs among different orders, probably as a result of adaptation to different living environments.

Meanwhile, the BLB-BRD-DMA-DMB-BF-TAP1-TAP2 cluster is conserved in crested ibis and Galliformes birds. The BLB encodes a class II β chain, and DMA/DMB are involved in the assembly of MHC class II peptides [42]. Similarly, the BF encodes a class I α chain, and TAPs encode the transporters associated with the process of loading peptides onto a class I molecule. Moreover, class I genes locating near TAP genes have also been found in Rat [43] and saltwater crocodiles [44]. It has been postulated that gene coevolution is present in the MHC region and such coevolution in the primordial MHC may have been responsible for the appearance of the antigen presentation pathways at the birth of the adaptive immune system [45, 46]. Therefore, we inferred that the close proximity of functionally related BF and TAP genes facilitate the coevolution of themselves.

In conclusion, recombination-based CSA was shown to be an efficient strategy for high-diversity highly polymorphic genomic region research, and could be a competitive alternative to traditional methods. With the CSA strategy, the crested ibis MHC was constructed in this study. Comparison analyses suggested fast evolution of MHCs among avian lineages. On the other hand, conserved MHC gene blocks in different vertebrate lines provided preliminary evidence to support the hypothesis that coevolution should have been responsible for the appearance of the antigen presentation pathways at the birth of the adaptive immune system.

## Methods

### Ethics statement

With the permission of the authorities, DNA samples were collected from peripheral blood of a female crested ibis in Yangxian County Reserve, Shaanxi Province, China. Blood sample collection was performed by professionals in the reserve. Ethical approval was granted by the Institutional Review Board on Bioethics and Biosafety in BGI, China (FT14035).

### Target sites selection

First, using tblastn, 41 protein sequences of chicken MHC genes (GenBank: AB268588.1) were aligned to the crested ibis genome (GeneBank: GCF_000708225.1). Homologous exon segments of 28 chicken MHC genes were found to be mainly distributed on 4 scaffolds of the crested ibis genome (S5 Fig). Second, one target site (500–1,000 bp in size) was chosen at every 30 kb region on the 4 scaffolds in the following priority order: homologous exon > intron > intergenic regions. The sequence uniqueness of the target site was tested by blastn with the database of the crested ibis genome. Totally, 17 target sites were selected and their locations are shown in S5 Fig and S1 Table.

### Fosmid library and PCR-based positive pools screening

A library of genomic DNA was constructed with the CopyControl HTP Fosmid Library Construction Kit following the manufacturer’s protocol (Epicentrue). Before recombination, the entire library was pre-screened to find the positive pools for each target site. The screening primers were designed on these fragments with the Primer3 tool (http://frodo.wi.mit.edu/primer3/; S1 Table). All primers were synthesized at Invitrogen. Primer specificity and annealing temperatures were tested on crested ibis genomic DNA. For the PCR template, 50 ng of DNA that were extracted from each pool was used. PCR amplification was performed using ABI9700 with the following parameters: 94°C for 5 min; 35 cycles of (94°C for 30 sec; annealing for 30 sec; 72°C for 30 sec); with a final extension at 72°C for 5 min. To further confirm the positive pools, the PCR products were also sequenced with the Sanger method.

### Construction of recombinant cassettes

To reduce the cost of the recombination step, ampicillin was used to replace blasticidin (Bsd) in the study [47]. The cassettes were produced by PCR using the *ampR* gene and a pair of 75 bp primers. The 75 bp primer consisted of the 50 bp homology arm of the targeted region and the 25 bp *ampR* amplification primer (S2 Table). The homology arm was chosen based on the following criteria: 1) G+C < 35 nt; 2) G+C difference between the F arm and R arm < 2 nt, to be in close proximity to the screening primer pair. An in-house PERL script was developed to perform the filter process. All forward primers were phosphorylated at the 5’ end to generate PO (O strands for hydroxyl), and the reverse oligos were not modified. Using the PMD-18T vector as the template of the *ampR* gene (TaKara), PCR amplification with Phusion DNA Polymerase (Thermo Fisher) was performed on ABI9700 with the following parameters: 98°C for 30 sec; 35 cycles at (98°C for 30 sec; 55°C for 30 sec; 72°C for 30 sec); and a final extension at 72°C for 5 min. The cassettes were purified from the PCR reaction using 1.5% agarose gel electrophoresis followed by use of a Qiagen Gel Extraction Kit.

### Recombinant procedure

Nedelkova’s vectors were used, and the recombinant procedure was performed as recommended by studies [22] with the following modifications. First, 25 μl aliquots from the PCR positive pools were grown in 1 ml of LB supplemented with tetracycline (tet, 5 μg/ml) and chloramphenicol (cm, 10 μg/ml) overnight at 30°C. A 30 μl overnight culture was diluted to 1.4 ml LB with tet (5 μg/ml) and cm (10 μg/ml) at 30°C for 2 h, followed by the addition of arabinose (Sigma A3256) and rhamnose (Sigma R3875) to 15% and growth at 37°C for 45 min. Second, the cells were centrifuged, washed twice with 1 ml ice-cold 10% glycerol and resuspended in 30 l ice-cold 10% glycerol. About 300–600 ng recombinant DNA was added to 30 μl of competent cells. For each electroporation, a pre-chilled 1 mm electroporation cuvette was used at 1350 V, with a 10 μF (Eppendorf Electroporator 2510). After electroporation, the cells were resuspended in 1 ml LB and incubated at 37°C for 1 h, and then plated on LB agar supplemented with 100 μg/ml ampicillin. The plates were incubated at 37°C for 18 h to 24 h. Third, 4–10 clones per recombinant cassette were incubated in 1ml LB supplemented with 100 μg/ml ampicillin and grown overnight at 37°C. PCR validation with screening primers was used to confirm the recombination success.

### Library construction and sequencing

The fosmid DNA from 41 clones was isolated with a Qiagen plasmid plus kit (Qiagen, Hilden, Germany) and sheared using a CavorisLE220 device (Covaris, Inc. Massachusetts, MA, USA). DNA fragments that were 500 bp, 2 kb and 5 kb in size were recovered. End-repair, A-tailing and paired-end adapter ligations were performed following the protocol in the kit NEBNext^®^ DNA Library Prep Master Mix Set for Illumina^®^ from New England Biolabs. All libraries were quantified by real-time PCR using a SYBR Fast Illumina Library Quantification Kit (KapaBiosystems). Next, every 16 libraries were pooled together with equal content. Paired-end sequencing (2x100 bp) was performed on an Illumina HiSeq 2000 following the manufacturer’s instructions. About 60,000 pair raw reads were generated for each library.

### Data filtering

To facilitate an accurate assembly, we performed a series of checking and filtering measures. The raw reads were filtered as follows: 1) Reads were trimmed 2 bp at the 5’end and 3 bp at the 3’end; 2) Reads with adaptors and with a small size were filtered; 3) Reads with >40% base quality below Q7 were filtered; 4) Reads with >10% “N” base were filtered; 5) The duplicate reads generated by PCR amplification in the library construction process were filtered; and 6) Reads with >30 bp mapped to either bacterial sequences in the NCBI BCT database or fosmid vector sequence were filtered.

### Short-read assembly and evaluation

Each fosmid clone was separately assembled with SOAP*denovo* [48]. We optimized assembly performance by varying the values of Kmer (K = 64…120) to get a longer scaffold N50. First, the pair-end reads from the 500-bp library were employed to assemble the contigs. Second, reads from 2 kb and 5 kb large insert-size libraries were mapped to the assembled contigs with *SOAPalign* and the exclusively mapped reads were used to construct scaffolds. Also, the gaps in scaffolds were closed using Kgf and GapCloser. Third, clones derived from the same recombinant cassette were assembled into one scaffold using 2 steps: 1) taking the longest scaffold of each clone together and determining the mutual positional relationship of these scaffolds by aligning them using MAFFT with the parameter "—clustalout", 2) merging the scaffolds into one second class scaffold using an in-house script, and in the regions covered by more than 1 clone, the assembly with a smaller gap was kept. Finally, with the same strategy, the second class scaffolds of 17 target sites were assembled to super-scaffolds. To evaluate the quality of assemblies, we first aligned our CSA sequence to Sanger and other sequence versions by BLAT and then we compared the SNP or short indel difference manually. The resulting MHC sequences were deposited in GenBank (accession number: KR995141—KR995143).

### Annotation

Homology and *de novo* prediction, as well as RNA-Seq data, were used to identify genes. First, the MHC region genes of chicken, zebra finch, mallard, peregrine falcon and pinnated grouse from NCBI were used to generate the corresponding protein sequences, which were then mapped to our crested ibis MHC sequence using tblastn with a cutoff E-value of 1e-5. All high-score segments were grouped into gene-like structures by genBlastA [49]. The intron-included homologous sequences with flanking 500 bp were aligned to the protein sequences using Genewise [50]. Second, genes were predicted using a *de novo* software Augustus [51] with model parameters trained from a set of high-quality homolog prediction proteins in the crested ibis genome. Third, with RNA-Seq data [23], the transcripts were clustered using TopHat [52] and Cufflinks [53], and then aligned to SwissProt/TrEMBL database [54] with a cutoff E-value of 1e-5. Finally, the identified genes were merged to form a comprehensive and non-redundant gene set following 3 criteria: 1) candidate genes clustered with a >100 bp genomic overlap; 2) one cluster per gene (precedence: homology-based model > RNA-seq model > *de novo* predicted model); and 3) if not met 2), the gene required a >30% aligning rate to a known protein in the SwissProt/TrEMBL database and at least 2 exons. TEs were identified using RepeatMasker with default parameters. tRNAs were identified using tRNAScan with default parameters. STRs were identified using TandomRepeatFinder. GC content was calculated in 500-bp sliding windows.

## Supporting Information

S1 Fig Insert size distribution of 41 sequenced clones. (TIF)

S2 Fig Ten assembly differences between Sanger and CSA. Each box shows one difference corresponding to Table 1.
(TIF)

S3 Fig The mapping of reads sequenced from WGS to CSA. Every uniquely mapped reads pair is shown as a semicircle. The reads pairs from different WGS libraries are shown with different colors (2 kb, green; 5 kb, blue; 10 kb, red; 20 kb, black). The gaps on the scaffold are marked as white blocks. The arrow indicates reads pairs which supporting the linkage of SC2 and SC3. RC means reverse and complementary.
(TIF)

S4 Fig **Alignment between CSA and Chen’s report at the extended BF/BL region (A) and BF/BL region (B and C).** Read depth on the CSA was calculated by mapping the short reads onto the CSA sequences. The predicted STRs and TEs are shown in green and red, respectively. The gaps on the scaffold are marked as white blocks. The annotated genes are shown beside the scaffolds.
(TIF)

S5 Fig Location of target sites on the crested ibis genome. The target sites are marked as blue diamond. The predicted gene or exon fragments are shown in green. The gaps on the scaffold are marked as black blocks.
(TIF)

S1 Table PCR primers used for positive pool screening. (XLSX)

S2 Table Oligos used for generation of the *ampR* selection cassettes. All forward oligos are phosphorylated at the 5’ end.
(XLSX)

S3 Table Assembly of clones at each recombination site. (XLSX)

S4 Table Comparison of crested ibis MHC genes with previous reports. (XLSX)

S5 Table Comparison of TEs between crested ibis MHC and chicken MHC-B. (XLSX)

S6 Table tRNAs in the crested Ibis MHC. (XLSX)
