# Supplementary figures and images for: Signatures of Crested Ibis MHC Revealed by Recombination Screening and Short-Reads Assembly Strategy
Source: PLoS One. 2016 Dec 20;11(12):e0168744. doi: 10.1371/journal.pone.0168744 (PMC5173252; doi:10.1371/journal.pone.0168744)

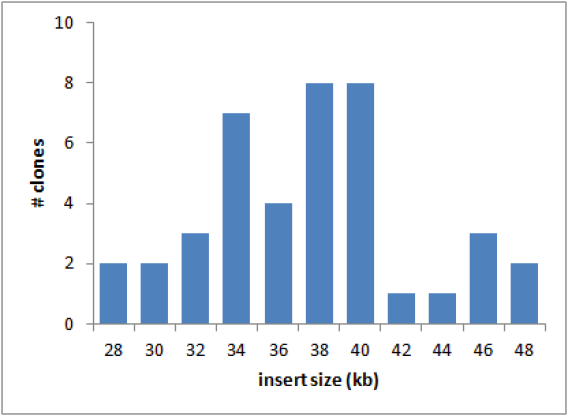

Supplement: S1 Fig — (TIF) [file pone.0168744.s001.tif]

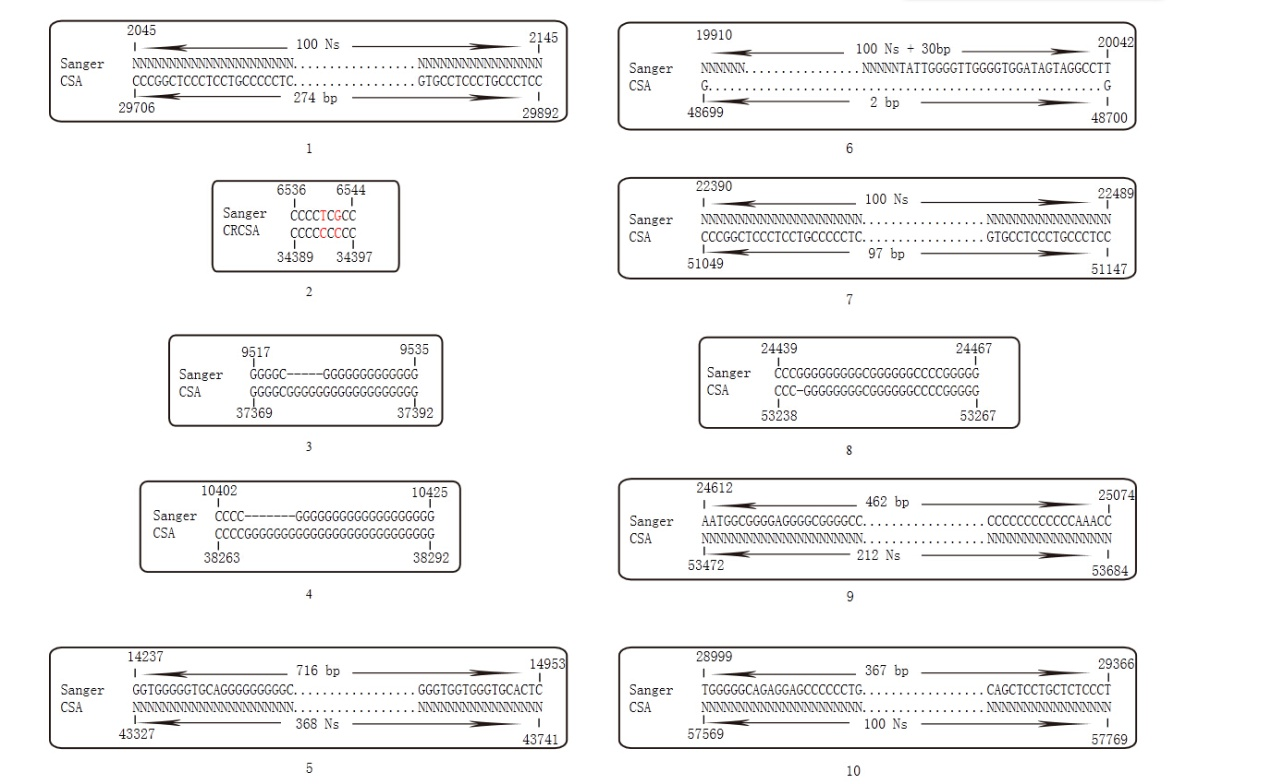

Supplement: S2 Fig — Each box shows one difference corresponding to Table 1. (TIF) [file pone.0168744.s002.tif]

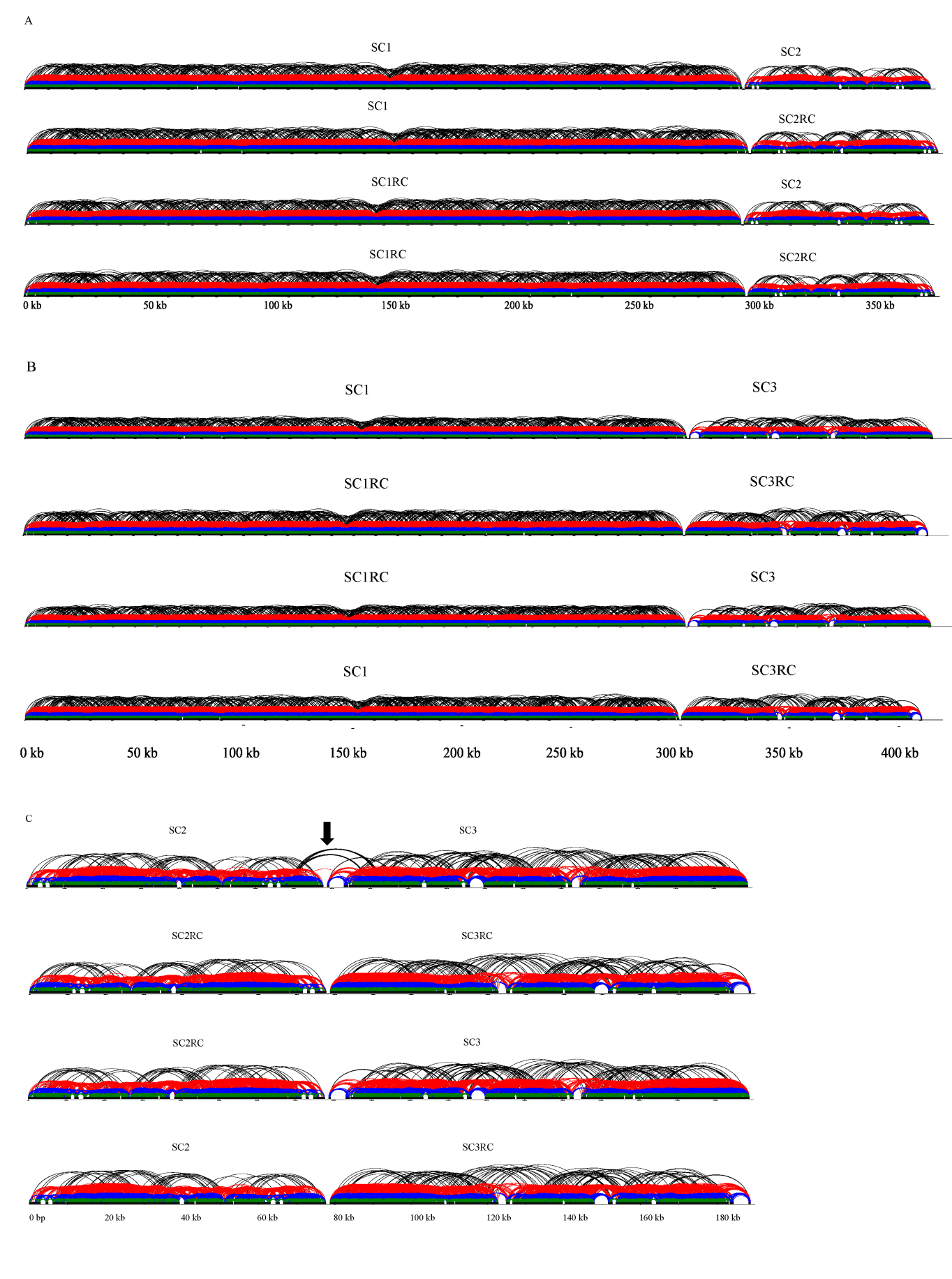

Supplement: S3 Fig — Every uniquely mapped reads pair is shown as a semicircle. The reads pairs from different WGS libraries are shown with different colors (2 kb, green; 5 kb, blue; 10 kb, red; 20 kb, black). The gaps on the scaffold are marked as white blocks. The arrow indicates reads pairs which supporting the linkage of SC2 and SC3. RC means reverse and complementary. (TIF) [file pone.0168744.s003.tif]

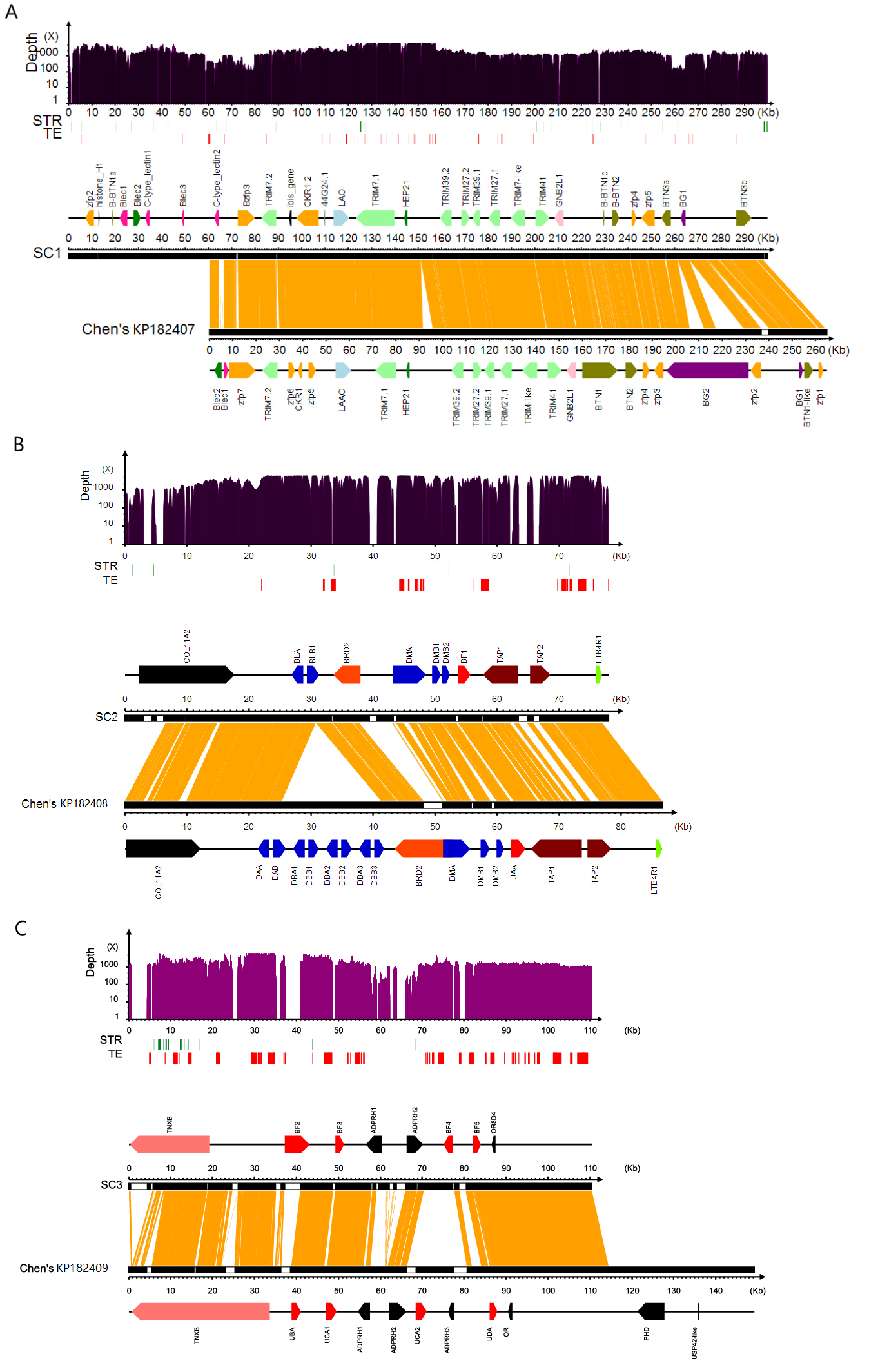

Supplement: S4 Fig — Alignment between CSA and Chen’s report at the extended BF/BL region (A) and BF/BL region (B and C). Read depth on the CSA was calculated by mapping the short reads onto the CSA sequences. The predicted STRs and TEs are shown in green and red, respectively. The gaps on the scaffold are marked as white blocks. The annotated genes are shown beside the scaffolds. (TIF) [file pone.0168744.s004.tif]

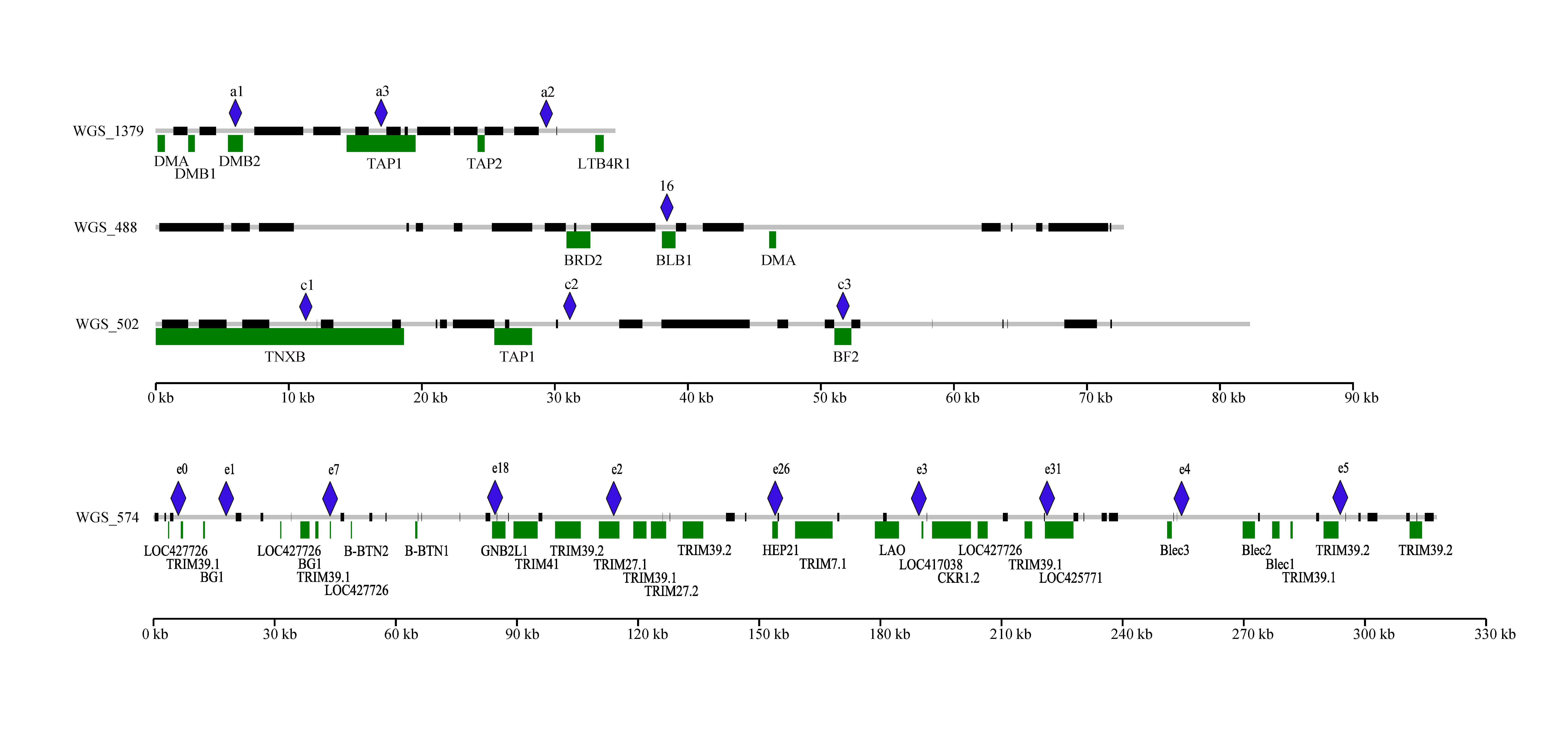

Supplement: S5 Fig — The target sites are marked as blue diamond. The predicted gene or exon fragments are shown in green. The gaps on the scaffold are marked as black blocks. (TIF) [file pone.0168744.s005.tif]
